# Immunology of Oral Squamous Cell Carcinoma—A Comprehensive Insight with Recent Concepts

**DOI:** 10.3390/life12111807

**Published:** 2022-11-07

**Authors:** Sowmya Samudrala Venkatesiah, Dominic Augustine, Deepika Mishra, Neethi Gujjar, Vanishri C. Haragannavar, Kamran Habib Awan, Shankargouda Patil

**Affiliations:** 1 Department of Oral Pathology & Microbiology, Faculty of Dental Sciences, Ramaiah University of Applied Sciences, MSR Nagar, Bengaluru 560054, India; 2 Department of Oral Pathology & Microbiology, Centre for Dental Education and Research, All India Institute of Medical Sciences (AIIMS), Delhi 110608, India; 3 College of Dental Medicine, Roseman University of Health Sciences, South Jordan, UT 84095, USA; 4 Centre of Molecular Medicine and Diagnostics (COMManD), Saveetha Dental College & Hospitals, Saveetha Institute of Medical and Technical Sciences University, Chennai 600077, India

**Keywords:** biomarkers, gene expression, immune profiling, immunology, immunotherapy, oral cancer, tumor escape, tumor microenvironment

## Abstract

This review aims to understand the concept of oral cancer immunology through the notion of immune profiling, immunoediting and immunotherapy, and to gain knowledge regarding its application for the management of oral cancer patients. Oral cancer is an immunogenic tumor where the cells of the tumor microenvironment play an important role in tumorigenesis. Understanding the mechanism of these modulations can help design immunotherapeutic strategies in oral cancer patients. This article gives an overview of immunomodulation in the oral cancer tumor microenvironment, with concepts of immune profiling, immunoediting and immunotherapy. English literature searches via Google Scholar, Web of Science, EBSCO, Scopus, and PubMed database were performed with the key words immunology, tumor microenvironment, cells, cross talk, immune profiling, biomarkers, inflammation, gene expression, techniques, immunoediting, immunosurveillance, tumor escape, immunotherapy, immune checkpoint inhibitors, vaccines in cancer, oral cancer, and head and neck cancer. Original research articles, reviews, and case reports published from 2016–2021 (n = 81) were included to appraise different topics, and were discussed under the following subsections. Literature published on oral cancer immunology reveals that oral cancer immune profiling with appropriate markers and techniques and knowledge on immunoediting concepts can help design and play an effective role in immunotherapeutic management of oral cancer patients. An evaluation of oral cancer immunology helps to determine its role in tumorigenesis, and immunotherapy could be the emerging drift in the effective management of oral cancer.

## 1. Introduction

Oral squamous cell carcinoma (OSCC) is characterized by extensive infiltration of immune cells, and is regarded as a highly immunogenic tumor [1]. The complex milieu of the tumor microenvironment (TME) comprises of an extracellular matrix (ECM), a variety of stromal cells, and immune cells which synchronize and interact with tumor cells. Examples include tumor-associated macrophages (TAMs), regulatory T cells (Tregs), cancer-associated fibroblasts (CAFs) and endothelial cells [2]. Macrophages, dendritic cells (DCs), neutrophils, myeloid derived suppressor cells (MDSCs), natural killer cells (NKs), and innate lymphoid cells are the innate immune cells, and the adaptive immune cell comprises of T cells and B cells [3]. Cross talk between the cells of the TME, ECM and the tumor cells is significantly responsible for fueling the tumor development [4,5]. In addition, the cancer-associated inflammation, more precisely chronic inflammation, involves inflammatory cytokines, chemokines and growth factors which could trigger DNA damage, tumor angiogenesis, and genomic instability, as well as the tussle between immunosuppression and promotion leading to tumorigenesis [6]. Furthermore, involvement of the -omic mechanisms plays a determinant role in extracting features describing the cellular and molecular heterogeneity of the TME and tumorigenesis [7]. However, evidence suggests that not all cells transform into cancerous cells; the credit for this goes to the process called “immunosurveillance”, or a more broadened term known as “cancer immunoediting”. The process of both curtailing and promoting the tumor development by the immune system is referred as cancer immunoediting. Cancer immunoediting broadly comprises three phases, namely elimination (immunosurveillance), equilibrium, and escape [8,9]. The concept of cancer immunoediting is widely accepted, and tumor immune escape is considered an emerging hallmark of cancer [10]. The response to this knowledge of the immune system in cancer has led to a paradigm shift in the management of cancer from conventional therapies such as surgery and radiotherapy to immunotherapy. The goal of immunotherapy is to target only the cancer cells, overcome the immunosuppression induced by a tumor and its microenvironment, and to increase successful response rates by tailoring personalized immunotherapies and predictive biomarkers [11,12]. This article provides an overview of the modulation between the immune system and the oral cancer microenvironment, immune profiling, immunoediting, and current and emerging immunotherapeutic strategies in oral cancer. This review aims to inspire clinical researchers to embrace immune-based research studies by providing an overview of the most recent findings in oral cancer; this will hopefully encourage clinical practitioners to recruit immunotherapeutic approaches, which will help to understand its clinical efficacy in the management of oral cancer patients.

## 2. Materials and Methods

Literature searches via Google Scholar, Web of Science, EBSCO, Scopus, and PubMed database were performed for the key words immunology, tumor microenvironment, cells, cross talk, immune profiling, biomarkers, inflammation, gene expression, techniques, immunoediting, immunosurveillance, tumor escape, immunotherapy, immune checkpoint inhibitors, vaccines in cancer, oral cancer, and head and neck cancer. Original research, reviews, case reports and short communications published in the English language were included (Total number of articles screened = 81). Two reviewers selected the articles and assessed them for information on cross talk between the immune system and tumor cells within TME, key biomarkers of tumor microenvironment and immune gene expression profiling. An attempt was made to understand the methods adopted in immune profiling, clinical use of immune profiles, immunoediting in oral cancer, and immunotherapy in oral cancer.

## 3. Immune Profiling of Oral Cancer

Distinctive changes to oral cancer staging have been made by *The American Joint Committee on Cancer (AJCC) Staging Manual* in the “Head and Neck” section of their 8^th^ edition [13]. Yet, in response to therapy and aggressiveness, it is observed that tumors of the same stage are heterogenous in nature [1]. An addition of immune contexture can provide information that can be equally effective or even superior to TNM staging in predicting progression and overall survival [14]. The genomic and molecular signatures of oral cancer and of the infiltrated immune system focus on identifying key biological molecules that could be linked to cancer development, risk assessment, screening, predicting recurrence, prognosis, and invasion/metastasis, and represent important determinants of immunotherapeutic responses [15,16,17]. The significance of immune profiling is attributed to the fact that patients in various molecular subgroups may respond impeccably to different treatment [14]. Furthermore, advances in technology have revolutionized immune profiling from interrogating a single marker by immunohistochemistry (IHC) to investigating enormous immune-related genes by sequencing [18]. This section of the article discusses the importance of profiling TME and tumor cells, their biomarkers, identifying immune-cell-associated genes and proteins, and the methods employed for immune profiling in oral cancer.

### 3.1. Cross Talk between Immune System and Tumor Cells within TME

The impact of the tumor cells strikes cause the cellular component of TME to secrete various cytokines and chemokines. This presents one of the prime pitfalls in the management of OSCC patients. An insight into these interactions can aid in determining the prognosis and effective development of therapies [19,20]. A critical role in carcinogenesis is exhibited by the macrophages or TAMs. The classically activated M1-type and the alternatively activated M2-type are the two subgroups of macrophages [21]. TGF-β1, interleukin8 (IL-8), tumor necrosis factor- α (TNFα), vascular endothelial growth factor (VEGF), epidermal growth factor (EGF), and matrix metalloproteinase (MMP), which are essential for tumorigenesis are some of the cytokines secreted by TAMs [22]. A study by Kubota et al. highlighted that T cell apoptosis and immunosuppression in OSCC patients is exhibited by CD163+ and CD204+ TAM subsets via IL-10 and PD-L1 [23]. A recent study by Haque AR et al. observed that EGF production by CD206+ TAMs promote proliferation and invasion in OSCC [24]. Cytokines TGF-β, IL-13, and IL-1 secreted by M2 macrophages are also known to promote tumorigenesis in OSCC [25]. Despite the central role played by antigen specific immunity and tolerance, DCs constitute a rare immune cell population within tumors. DCs present tumor-associated antigens on major histocompatibility (MHC) molecules, provide costimulation and soluble factors to shape T cell responses in the TME [26,27]. DCs are of two types, namely myeloid dendritic cells (mDC) and plasmacytoid dendritic cells (pDC) [28]. A study by Zhang et al. found that accumulation of IL6 in the saliva of oral cancer patients was due to immunodeficiency of DCs [29]. In another study by Xiao et al., CD103+ T and DCs, which are well known for their role in antitumor immunity, indicated a favorable prognosis in oral cancer [30]. According to a study by Han et al., the TNF-α/NF-κB/CXCR-4 pathway promoted proliferation and invasion in OSCC and was strongly associated with significant rise in tumor-infiltrating pDCs [28]. NK cells are cytotoxic effectors, and their target is lysis of cancer stem cells and undifferentiated or poorly differentiated tumors. NK cells regulate the function of other cells through cytokines and chemokines, and mediate direct and antibody-dependent cellular cytotoxicity (ADCC) against tumors. The two subsets of NK cells are CD56dimCD16+ and the CD56brightCD16−/+ [31,32]. Triggering CD16 surface receptors leads to the secretion of cytokines, predominantly interferon-γ (IFN-γ) and TNF-α, by NK cells [33]. The negative regulation of the adaptive immune system by inducing IL-2, retinoic acid and TGFβ is essentially carried out by Tregs. The two groups of Tregs include thymus-derived Tregs (tTregs) and periphery-derived Tregs (pTregs). The expression of the transcription factor Forkhead box P3 (FOXP3) is responsible for accurate functioning of Tregs [34,35]. A study by Lee et al. suggests that CCR6+ Treg cells are recruited and retained in OSCC inflammatory milieu via CCL20/CCR6 axis from the inception of tumor progression [36]. Aggarwal et al. assessed phenotypic and functional characteristics of Tregs, and observed that the CD4+FoxP3+ subset showed skewed expressions of IL-2, IL-10 and IL-35 in OSCC patients [37]. CD4+ helper and CD8+ cytotoxic T lymphocytes are also known to express high levels of PD-1 in OSCC [38]. Apart from the aforementioned interactions, enormous modulations occur in the oral cancer TME, such as the high-endothelial venules. These modulations were correlated with: an increased number of CD3+ T cells and CD20+ B cells with higher levels of the chemokines CXCL12 and CCL21, and lower levels of CCL20 [39]; CAF activation induced by TGF-β released by oral carcinoma cells [40]; the involvement of ECM molecule tenascin-C in CD11c+ myeloid cell; CCL21 in lymphatic endothelial cells via integrin 1β9α [41]; and increased secretion of cytokines IL-1β, VEGF and IL17 in blood neutrophils [42].

### 3.2. Key Biomarkers of TME

#### 3.2.1. Immune and Non-Immune Cell Markers

Significant variations in the number of T cells, MDSCs, TAMs, and many other immune and non-immune cells are reported in OSCC [43,44,45]. This profiling of the cells with biomarkers can not only help to predict diagnosis and prognosis, but can also determine factors for proper immunotherapeutic approach in oral cancer patients [46]. Some of the signature immune cell biomarkers used in OSCC studies include CD11c, CD80 and HLA-DR for M1 TAMs, while CD163, CD11b, CD206 and MRC1 are used for M2 TAMs detection. Although not specific, CD68 is used as a pan macrophage marker [47]. A variety of markers are used for DC detection, which includes S100, CD1a, CD83, CD207, CD208, CD80, CD11c, CD86 and HLA-DR, but widely accepted markers are CD1a for immature DCs, and CD83, which has been shown to be expressed on activated and mature DCs [48]. A study by Taghavi et al. assessed the prognostic value in OSCC by activated NK cells using CD57 as a marker [49]. CD3 is pan T cell marker and CD8 is cytotoxic T cell marker [1]; a study by Mukherjee et al. demonstrated that quantifying CD3 and CD8 cell density helps to predict progression in gingivo-buccal OSCC [50]. T helper cell markers are CD4 [1] and Tregs, which is a subset of CD4+ cells, and multiple markers are used for the identification of OSCC. This involves CD25, CD127, cytotoxic T lymphocyte-associated antigen 4 (CTLA-4), lymphocyte activation gene-3 (LAG3) and forkhead/winged-helix transcription factor box P3 (FOXP3). However, the most accepted markers for Tregs are CD4+ CD25+ FOXP3+, and CD4+ CD25+ CD127low [34]. Pan B cell markers are CD19 and CD20 [1], and a study by Lao et al. revealed that CD19+ B cells were a favorable prognostic factor in oral tongue squamous cell carcinoma (OTSCC) [51]. Other cells include: MDSCs, which do not exhibit uniform markers but have commonly been identified to express CD33 and CD11b [52]; CAF, which express α SMA [51]; and CD31 and CD34, which are expressed on endothelial cells [53]. Table 1 represents various cells of the TME with their biomarkers.

#### 3.2.2. Inflammatory Biomarkers

Evidence suggests that chronic inflammation plays a prominent role in the development of OSCC [54]. The production of inflammatory mediators occurs to activate the immune system against the tumor, but inflammatory mediators can even stimulate tumor growth and could be utilized as potential biomarkers [55,56]. The role of systemic inflammatory response (SIS) is widely studied in cancers. Neutrophil-to-lymphocyte ratio (NLR), lymphocyte-to-monocyte ratio (LMR), platelet-to-lymphocyte ratio (PLR) and C-reactive protein (CRP), are the common biomarkers employed to assess SIS [56]. In a study by Hasegawa et al., the prognostic values of NLR, LMR, and PLR were investigated, and they found that high NLR was associated with poor prognosis. They concluded that it might be used as a potential independent prognostic biomarker in Japanese OSCC patients [57]. NLR is also known to be significantly associated with disease-specific survival in young patients with OSCC [58]. CRP is an early marker for inflammation, and it was seen that the preoperative serum CRP level effect was more prominent in oral cancer and was a prognosticator in OSCC [59]. The nuclear factor kappa-beta (NF-κB) plays a key role in the inflammatory process, and is linked to carcinogenesis [56]. The receptor for activated C kinase 1 promotes M2 macrophage-like polarization by regulating NF-kB, and progresses OSCC [60]. Modulation of the immune response is prominently carried out by the cytokines, which are broadly classified into two groups, namely proinflammatory (IL-1, IL-6, IL-8, TNF-α, and TGF-β) and anti-inflammatory (IL-2, IL-12, IL-4, IL-10, and IFN-γ) cytokines [56]. In a meta-analysis by Chiamulera et al., salivary cytokines as biomarkers were evaluated in oral cancer, and it was seen that IL-8, IL-6, TNF-α, IL-1β and IL-10 salivary levels were significantly higher in oral cancer patients [61]. According to Ferrari et al., IL-6, IL-8, and TNF-α were cytokines frequently investigated as candidates for OSCC biomarkers, and were present at higher concentrations in the saliva of OSCC patients [62]. Cyclooxygenases are enzymes which convert arachidonic acid into prostaglandins; a strong association between the expression of cyclooxygenase (COX-) 2 and chronic inflammation is seen in the initiation of carcinogenesis [56]. A study by Thomas et al. observed a significant increase in COX-2 expression from well to poorly differentiated OSCC [63]. Cell migration and the regulation of the level of cytokines at the site of inflammation is carried out by MMP, and their roles as biomarkers were analyzed by many researchers in OSCC [56]. A systematic review and meta-analysis by Miguel et al. showed that MMP-1, 2, 3, 7, 9, and MT1-MMP played a key role in lymph node metastasis and MMP 9 could serve as a prognostic biomarker of OSCC [64]. According to a study by Hsiao et al., when biomarker MMP1 was analyzed in dried saliva spot samples, they detected elevated salivary MMP1 levels in OSCC [65].

### 3.3. Immune Gene Expression Profiling

The discovery of the gene signature of the tumor cells and their microenvironment, especially with a pooled gene panel, can improve the accuracy of determining the heterogeneity of the tumor, risk stratification, immunotherapeutic interventions, and prognosis [66,67]. The requirement of minimal tissue is an advantage of gene expression profiling, as the material obtained from tissue specimens are usually limited [14]. In a study by Basu et al., numerous novel differentially hyper and hypomethylated immune genes were detected (LXN, ZNF154, ZNF577, ZSCAN31, PTPN22 and RUNX1.) through a genome-wide DNA methylation profile. They concluded that the TME of OSCC exhibited a strong immune component, which could serve as a potential molecular marker in identifying risk and prognosis [68]. In another study by Li et al., gene expression profiling was done to analyze the cytokines and chemokines responsible for lymph-node neogenesis. They identified a IL7, LTB and CXCL13 upregulated gene set, which was shown to be correlated with human oral cancer-associated tertiary lymphoid structures and could serve as future prognostic markers [69]. A study by Meehan et al. on the immune gene expression profile of OTSCC showed 24 genes (ARG1, CCL11, CD79 and IL6) which discriminated against more aggressive cases and were consistent with proinflammatory phenotype [70]. In a study by Chatzopoulos et al., RNA expression profiling of OTSCC showed upregulation of CTLA4, TIGIT and PD-L2. IDO1, TGF-β, B7-H3 and PD-L1 were known to be involved in inhibitory tumor mechanisms [71]. An immune score-based gene panel (ARMH1, F2RL2, AC004687.1, COL6A5, AC008750.1, RAB19, CRLF2, GRIP2, and FAM162B genes) was done to identify the risk stratification and predict prognosis in OSCC patients by Huang et al. They concluded that this profiling could help to determine the best course of treatment in individual OSCC patient [72].

### 3.4. Methods Adopted in Immune Profiling

**Figure 1:**
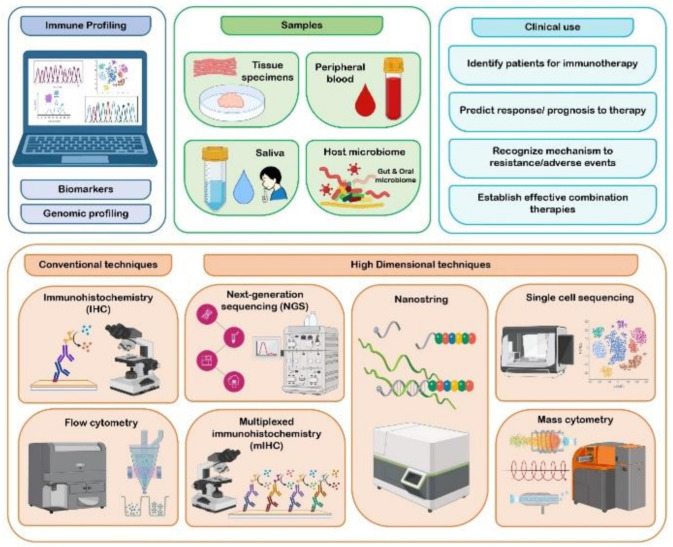
Depiction of methods used in immunoprofiling.

Immune profiling technologies can be broadly categorized into transcriptomic-based and proteomic-based technologies. The conventional immune profiling technologies, which included IHC and fluorescent-based flow cytometry, possessed many limitations, such as ineffectiveness in determining the degree of heterogeneity or the complexity and depth of immune phenotypes. These limitations were overcome by high dimensional technologies, which included next generation sequencing techniques (NGS), single-cell sequencing, NanoString nCounter technology, multiplex IHC (mIHC), and mass cytometry [14,73]. NGS technology comprises whole-genome sequencing (WGS), whole-exome sequencing (WES), RNA sequencing, miRNAs profiling and T cell receptor (TCR) sequencing, and is being exploited to investigate genomic and epigenetic analysis [14,74]. In a study by Goertzen et al., the identification of genes responsible for recruitment, invadopodia and invasion of neutrophils was achieved through NGS platform, which was applied to TNFα-treated OSCC cells [75]. The emergence of the single-cell genomic technique is a powerful tool for dissecting cancers at the resolution of individual cells, but this platform is still at the infancy stage, requiring the optimization of technique and protocols [17,76]. NanoString nCounter is a high-throughput technology, primarily built for the analysis of gene expression. It possesses an advantage of rapidity and technical simplicity, as it does not require amplification, cDNA or library production, in comparison with NGS [77]. NanoString nCounter technology was effectively utilized in OTSCC studies to detect immune-related genes and for RNA expression profiling [70,71]. mIHC allows for the detection of multiple markers on a single tissue section simultaneously [78]. Qiao et al. used mIHC staining to analyze PD-L1, TILs (CD8+ T cells and FOXP3+ Tregs) expression in the TME of various oral diseases, including OSCC, and were able to accurately visualize various immune cells harboring complex immune phenotypes [79]. Techniques such as mass cytometry, combination of mass cytometry with flow cytometry (known as CyTOF), and mass cytometry-based mIHC are also employed in the immune-profiling of cancers [73]. The methods used have been depicted in Figure 1.

### 3.5. Clinical Use of Immune Profiles

**Figure 2:**
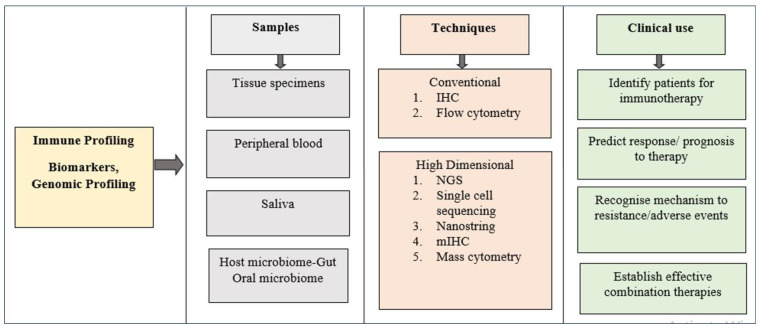
Represents various samples, techniques, and clinical use of immune profiling in cancers including OSCC.

An immune response to the tumor is usually unique to the TME, and understanding these responses through various biomarkers, genomic profiling and technologies may yield insights into the prognosis of the tumor, based largely on immunotherapeutic interventions. Immune profiles help to select patients who would benefit from immunotherapies in clinical set ups. For instance: Immunotherapeutic strategies with anti-programmed cell death-1 (anti-PD-1) and anti-programmed cell death ligand-1 (anti-PD-L1) antibodies play a crucial role in the treatment of head and neck SCC (HNSCC), including OSCC [80]. There are various studies being conducted by utilizing PD-1 and PD-L1 as biomarkers to understand their therapeutic efficacy in HNSCC [81,82,83]. A study by Foy et al. analyzed HPV-negative OSCC immune microenvironments in those who had never been smokers or drinkers through multiple approaches, which included genomic, protein expression and IHC on biomarkers CD3, CD4, CD8, IDO1, and PD-L1. They suggested a strong rational for immunotherapy, especially by targeting PD-L1 and IDO1 with pembrolizumab drug [84]. Shayan et al. showed that combination therapies with the addition of a PD-1 inhibitor to the anti-EGFR antibody cetuximab and toll-like receptor agonist (TLR8) motolimod could augment the antitumor response in HNSCC [85]. By contrast, in another study by Shayan et al. an adaptive resistance to anti-PD1 therapy was reported, which they suggested was due to the upregulation of T cell immunoglobulin mucin (Tim)-3 [86]. Hence, identifying and selecting the right set of patients is important in immunotherapeutic approaches, which is discussed in later section of the article, and immune profiling plays a key role in identifying, predicting response and resistance to immunotherapies, and helps in designing effective combination therapies for patient benefit. Figure 2 represents various samples, techniques, and clinical use of immune profiling in cancers including OSCC.

## 4. Immunoediting in Oral Cancer

**Figure 3:**
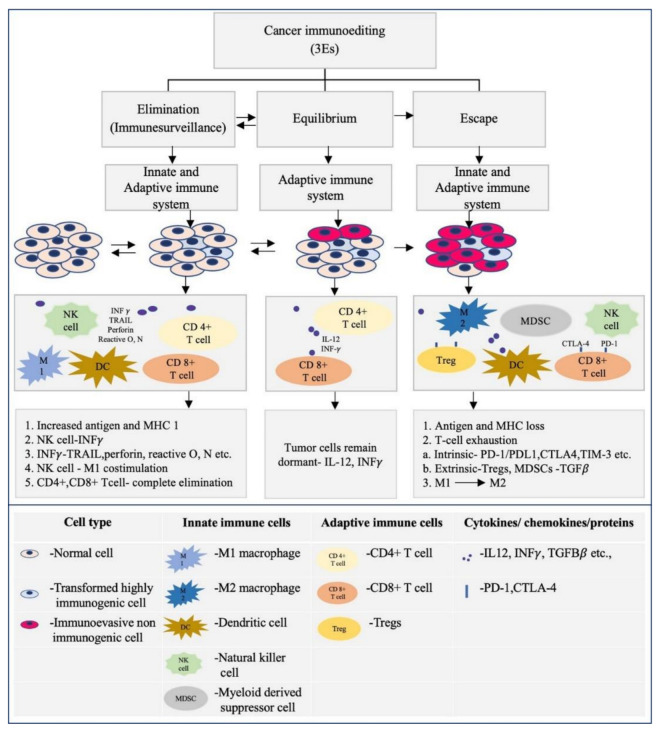
Represents the “3 Es” concept of cancer immunoediting in HNSCC/OSCC.

Immune system components are potentially endowed with dual function of both tumor progression and suppression, and can be considered a double-edged sword in cancer treatment [87,88]. This concept of cancer immunoediting has enormously evolved in the last few decades, with the goal of understanding the role played by the cells of the immune system in the development of cancer, and reversing its progression with the advent of immunotherapy [89]. Cancer immunoediting is broadly divided into three phases. Cancer immunosurveillance, or the elimination phase, is one phase, wherein the immune system eliminates the tumor cells, which is triggered by the acquisition of tumor-specific and tumor-associated antigens [90]. Figure 3 represents the “3 Es” concept of cancer immunoediting in HNSCC/OSCC.

### 4.1. Mechanism of Cancer Immunoediting

**Figure 4:**
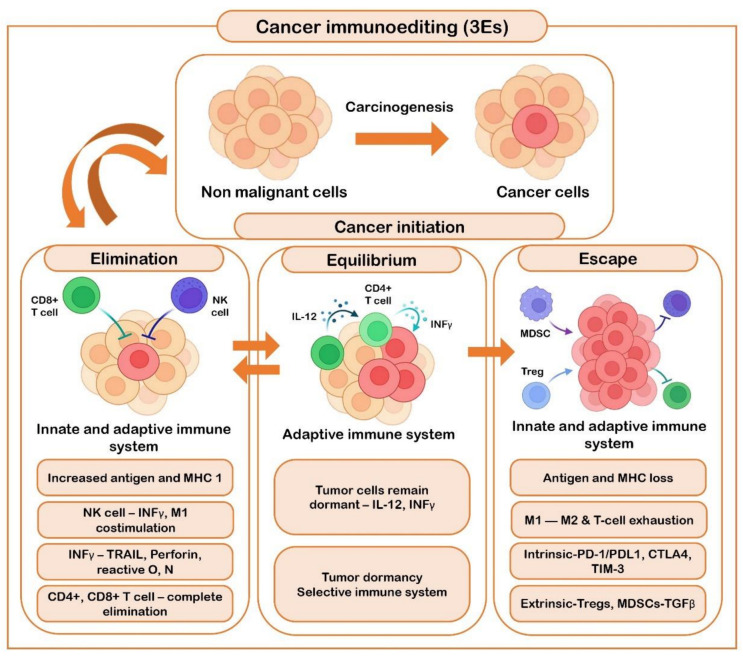
Represents the role of the immune system in carcinogenesis and cancer initiation.

The elimination phase of the immunoediting process is complex in nature, and characterized by the response of the innate and the adaptive immune system [91]. Dying tumor cells release cytokines type I IFNs, and the surface of the tumor cells is composed of ligands such as MICA/B and H60, which activate the immune system cells by binding to the receptors. In turn, CD4+T and CD8+T cells promote the coordination between the innate and adaptive immune response [92]. The second and the longest of the immunoediting process is the equilibrium phase, where tumor cells remain in the dormant stage maintained by IL-12, T cells, and IFN-γ factors. In this phase, the adaptive immune system plays a major role; there are two possible outcomes of this phase: either tumor regression and elimination, or tumor progression [89,92]. The most widely studied phase of cancer immunoediting is the escape phase, which is often the result of T cell exhaustion. This exhaustion is due to a T cell intrinsic mechanism which involves a PD-1/PD-L1 pathway and immunoregulatory receptors such as CTLA-4, TIM-3, and LAG-3, and a T cell extrinsic pathway mediated by Tregs and MDSCs through the secretion of cytokines such as TGFβ [91]. The crosstalk between hypoxia/hypoxia inducible factor (HIF)-1α and the immune cells contributes to tumoral immune escape in many tumors, including OSCC. This was found to be either due to the recognition by NK cells and cytotoxic T lymphocytes or the hypoxic stress. HIF-1α is known to induce immune-suppressive molecules such as IL-10 and TGFβ, leading to the differentiation of TAMs into M2 macrophages in order to suppress anti-tumoral activities. HIF-1α is also known to orchestrate the CD4+ and CD8+ T cells with respect to their survival, apoptosis, and cytokine secretion [93]. A study by Kanicka et al. found an increased immunoexpression of PD-L1, which is associated with increased tumor CD163+ macrophages. They showed that OSCC may evade the host immune system by PD-L1 immunoexpression not only on epithelial cells, but on infiltrating cells too. They also discussed on the concept of immunoediting where PD-L1 is implicated in tumor immune escape by inducing apoptosis in activated antigen-specific CD8+ cells [94]. Poor effector T cell trafficking is also known to be responsible for the immune escape in HNSCC [95]. According to Sanchez et al., CD68+ and CD163+ TAM infiltration was significantly associated with high expression of PD-L1, suggesting a link between TAM infiltration and immune escape in OSCC [96]. Figure 4 represents the role of the immune system in carcinogenesis and cancer initiation

### 4.2. Implication of Cancer Immunoediting in Oral Cancer Immunotherapy

The key pathways which operate the immunoediting process in its three phases can help design the most appropriate immunotherapies and combat immunotherapeutic resistance in a safe manner [97,98]. Various modes of immunotherapy, such as check point inhibitors, monoclonal antibodies, and vaccinations based on the framework of the immunoediting process, are being developed, and HNSCC/OSCC, is an excellent target for an immunotherapeutic approach [99,100]. Cramer et al. have discussed that exposure to a PD-1 inhibitor prolongs survival in recurrent/metastatic HNSCC, which includes OSCC, as proven by randomized phase III trials [101]. Mei et al. have also discussed various clinical trials either by monotherapy or combination therapy, which use checkpoint inhibitors targeting the immune checkpoint molecules such as CTLA-4, PD-1, TIM-3 and LAG-3. This has been shown to be a promising approach in HNSCC [102]. Nevertheless, it has also been observed that tumors in the head and neck region develop resistance to immunotherapy. Lee et al. have mentioned that resistant tumor clones in HNSCC which are left behind in immunoediting remain resistant to T cell killing and T cell-based immunotherapy, but are sensitive to NK cell-based immunotherapy; in such patients, a combination of T cell and NK cell-based immunotherapy may benefit them [103]. From the above-mentioned studies, it can be inferred that the immunotherapeutic approach, either through monotherapy or combination, both in research and clinical settings depends upon the understanding of the immune mechanism involved in cancers. In this regard, the immunoediting concept has tremendously helped researchers to gain knowledge on the immune mechanism involved in oral cancer tumorigenesis, understand the concept of optimal immunotherapy and immune resistance to therapy, and develop novel immunotherapeutic strategies for patient benefit.

## 5. Immunotherapy in Oral Cancer

The twenty-first century has resurfaced the concept of cancer immunotherapy with the advent of novel technology, molecular and biochemical advances, and experimentation on knockout mouse models [104]. Immunotherapies are broadly classified into active and passive immunotherapy [105]. Active immunotherapy gives a lasting response and is achieved by the direct stimulation of an immune system, with tumor cells as the target. Oncolytic vaccines, NK cells, DC, and cytotoxic T cells are commonly employed in active immunotherapy. Passive immunotherapies often produce specific short-lived response, targeting the cell surface receptors, and include monoclonal antibodies [99,105]. This section of the review discusses different categories of immunotherapeutic approaches and emphasizes immune checkpoint inhibitors, which unleashes a powerful T cell response and are commonly employed in HNSCC, and monoclonal antibody-based immunotherapy and cancer vaccines, which focus on prophylaxis and therapeutics. Lastly, the emerging approaches in oral cancer immunotherapy are discussed.

### 5.1. Immune Checkpoint Inhibitors (ICIs)

The therapeutic landscape in oncology is transformed by agents that target the interactions between PD-1/PDL1 and CTLA-4. These key immune regulatory pathways are targeted by ICIs [106]. A restrained T cell-mediated antitumor response is unleashed by ICIs. Although T cells are considered the keystone of ICI therapy, they also activate other cells of the innate and adaptive arms, which orchestrate a successful antitumor response [107]. ICI therapy is known to improve the overall survival in HNSCC patients. The primary cause of recurrence and metastasis in HNSCC/OSCC is due to tumor-induced immune evasion, which is partially mediated by T cell-suppressive immune checkpoint. ICIs enhance T cell activity and facilitate endogenous anticancer activity by removing the inhibition signals [108]. The first ICI drug approved by the FDA for HNSCC therapy was an anti PD-1 agent Nivolumab, in 2016. Currently, PD-1 drugs nivolumab and pembrolizumab, PD-L1 drugs atezolizumab, durvalumab, avelumab, and CTLA-4 ipilimumab and tremelimumab are tested in the treatment of HNSCC [108,109]. In a study by Estremera SD et al. on a preclinical mouse model of HPV+ oral cancer, the therapeutic efficacy of antiPD-1 immunotherapy was enhanced by targeting interferon signaling and CTLA-4 [110]. A study by Kim et al., which comprised 15 cases of SCC of the oral cavity and oropharynx, using ICI therapy with pembrolizumab or nivolumab, showed a promising efficacy in patients with recurrent and metastatic HNSCC [111]. In an open-label, non-randomized, multi-arm, phase 2 trial carried out by Sacco AG et al., combination therapy with PD-1 inhibitor pembrolizumab and EGFR inhibitor cetuximab showed an overall response rate of 45%. The response rates exceeded those of pembrolizumab (16–18%) or cetuximab (6–13%) monotherapy. The study consisted of platinum-resistant or platinum-ineligible recurrent or metastatic HNSCC patients, and included 45% participants with oral cavity as the primary tumor site [112]. Immune-related adverse reactions, toxicities, and resistance are common in ICI therapy [105,108,112] which demands for comprehensive work in the selection of patients.

### 5.2. Targeted Monoclonal Antibodies

Human or murine antibody components that bind to tumor-associated antigens lead to ADCC help in the production of monoclonal antibodies. An antibody against EGFR is the archetype which is therapeutically used in this group [104]. Depending on the targeting mechanism, the drugs that target EGFR are classified as monoclonal antibodies against EGFR, which includes cetuximab and nimotuzumab. Proliferation and migration of cancer cells are regulated by the EGFR protein through the ubiquitin/proteasome pathway. Numerous clinical trials are being carried out with drugs gefitinib, erlotinib and afatinib in the management of OSCC and HNSCC. These drugs belong to the EGFR tyrosine kinase inhibitors group [113,114]. Cetuximab is effectively used to treat locally advanced, recurring OSCC and patients with distant metastasis. FDA approval was also achieved to treat HNSCC with cetuximab in conjunction with radiotherapy. Combination of nimotuzumab and chemoradiotherapy is also known to be efficacious in treating OSCC [114]. EGFR is inhibited by tyrosine kinase inhibitor gefitinib, which has shown efficacy in the treatment of metastatic and recurrent OSCC. Gefitinib is also used in conjunction with paclitaxel, but its further application is limited due to toxicity [114,115]. The drugs afatinib and erlotinib inhibit EGFR1 phosphorylation and impede the growth of HNSCC cells. Afatinib also radiosensitizes HNSCC cells by targeting cancer stem cells [116]. Angiogenesis is promoted by VEGF and its receptors, which are known to show a high expression in OSCC [113]. Monoclonal antibodies such as bevacizumab, and multi-kinase inhibitors such as sorafenib, aflibercept and vandetanib, are two groups of VEGF inhibitors which are under various stages of investigations [114]. In a study by Yoshida et al., a reduction in growth in OSCC xenografts was observed by peritumoral bevacizumab injections [117]. Ganjibakhsh M et al. demonstrated aflibercept reduced the migration rate of cells in comparison to bevacizumab. They also signified that a combination of aflibercept with conventional surgical treatments can be effectively used in the treatment of OSCC [118].

### 5.3. Oral Cancer Stem Cells and Escape from the Host Immune Surveillance

Cancer stem cells (CSCs) can induce tumors in immunocompromised mice models. It has been observed that tumors without CSC fail to stimulate tumor formation; this concept clearly suggests the tumor induction potential of CSCs in the presence of immunosurveillance. A basic virtue of CSCs is to evade the host’s immune system, which is substantiated by the fact that non-CSCs can induce tumors in a more immunocompromised mouse [119]. Immune evasion mechanisms should be thoroughly evaluated to understand the mechanisms responsible, as these can be exploited to improve tumor immunotherapy. These mechanisms should be analyzed in immunocompetent or partially immunocompromised mice, as only this will reveal the true quantity of CSCs in the tumor. To conclude, CSCs are promising targets for tumor immunotherapy.

### 5.4. Cancer Vaccines

Cancer vaccinations are primarily employed to augment antigen-specific CD4+ and CD8+ T cells, which in turn help in the recognition and eradication of tumor cells. Antigen-presenting cells (APCs) play a key role, in that they persuade a robust antigen-specific T cell response by vaccines. Vaccine targets could be tumor-associated antigens (TAAs) or tumor-specific antigens (TSAs), such as viral antigens or neoantigens that emerge from single nucleotide variants and indels [120]. Vaccinations can be accomplished with single antigens, such as peptides and DNA/RNA, or multiple antigens, such as pulsed dendritic cells or whole cells [105]. Peptide vaccination is the most commonly applied technology and is considered safe, but the limitation is that abundant time is required for vaccine generation [120]. Zolkind et al. demonstrated that prophylactic vaccination with a mutant ICAM1 neoantigen-derived single long peptide (SLP) may help to produce a remarkable T cell response and protect against tumor development through their study on murine syngeneic oral carcinoma cell lines [121]. Nucleic acid-derived DNA and RNA vaccines are easier to synthesize than peptide vaccines. Plasmid DNA derived from bacteria as antigens are used to make DNA vaccines. Along with this, IL-2 and granulocyte/macrophage colony-stimulating factor immune stimulatory molecules are often used with DNA vaccines. Stability, the easy presentation on MHC-I and MHC-II of APCs and the robust activation of CD4+ and CD8+ T cells are some of the advantages of DNA vaccines. RNA vaccines do not integrate into the genome, and are delivered only into the cytoplasm of APCs, unlike DNA vaccines which need to penetrate the nuclear membrane. mRNA is commonly employed in RNA vaccines, but recently RNA replicons have also been explored [105,122]. Numerous complete and pre/active vaccine trials are conducted in HNSCC. HPV-antigen-related pre/active vaccine trials include NCT03821272, NCT02002182, NCT02865135, NCT03260023, NCT03418480 and NCT04369937. NCT02544880, NCT04247282 and NCT0368919 are the TAA-related vaccine trials, NCT02999646 is the cellular vaccine trial, and neoantigen vaccine trials carried out in HNSCC are NCT03568058 and NCT04266730 [119]. The effects of check point inhibitors are known to be accelerated by cancer vaccines broadening the approach of combination therapies [119]. In a study by Kumai et al. on a tumor mouse model, a combination design with a peptide vaccine, toll-like receptor agonist, and OX40/CD40, stimulation was seen to produce an effective antitumor CD4+ T cell response [123]. These are some of the combination therapeutic strategies incorporated to empower the vaccination efficacy.

### 5.5. Other Therapies and Advances

Adoptive T cell therapies are the latest trend which is used to fight HNSCC. These cell-based therapies are predominantly known to act on hematologic cancer. In this approach, T cells are genetically engineered and later primed to patient-specific antigens, leading to regression of the tumor. Activated cytotoxic T lymphocytes (CTLs) play a key role in this design of therapy. The most successful type of adoptive cell therapy is chimeric antigen receptor T cells (CAR-T) therapy. Numerous clinical trials are being conducted on solid tumors with the T cell therapeutic approach after its success in hematologic malignancies. [124,125]. A recent review by Muzaffar et al. mentioned a study where a promising result of 100% one-year overall survival, which was observed in 5 patients with advanced HNSCC who were treated with adoptive immunotherapy using ex-vivo-activated CTLs. HPV-16 E7 T cell receptor engineered T cells (NCT03912831) to determine the efficacy of KITE-439, a combination approach with an infusion of autologous tumor-infiltrating lymphocytes (LN-145/LN-145-S1) and recombinant IL-2 (NCT03083873) are being further investigated in the management of recurrent and metastatic HNSCC [125]. Activation of the immune system by cytokine therapy has been a major contributor to current clinical cancer research. Unfortunately, monotherapy with cytokines is often associated with dose-related toxicities. Hence, they are explored with other approaches such as anticancer vaccines, check point inhibitors and monoclonal antibodies, which are cancer-directed to elevate ADCC or sustain anticancer efficacy [126]. Apart from these combination therapies with TLR8, agonist motolimod with the EXTREME regimen (platinum-carboplatin or cisplatin/fluorouracil/cetuximab) [127], costimulatory agonists, such as OX40, CD40L, CD137, STING, IDO1, and STAT3 inhibitors with ICI therapy [128], adjuvant dendritic cell vaccine against p53, TriAd vaccine targeting brachyury, mucin-1 and carcinoembryonic proteins comprising of adenoviral vaccines are in various stages of clinical trials in the treatment of HNSCC [124,129].

## 6. Conclusions

Cancer treatment is being revolutionized in the current era due to the success of novel anticancer immunotherapies. Clinically durable responses in oral cancer could be elicited through immunotherapies. Unfortunately, the modest response rate to immunotherapeutic approaches in oral cancer calls for more comprehensive work to improve the outcomes. The linchpin for immunotherapy is selecting the right set of patients. Precision immunotherapy with the involvement of predictive markers could help overcome some of these limitations. This can be better achieved by the clear understanding of the fundamentals of immunomodulatory mechanisms within the oral cancer TME, and by having a knowledge on the immunoediting process in oral cancer. The advent of novel technologies and translational research has also revolutionized the immunotherapeutic approach in oral cancer patients. However, it is observed that most of the immunotherapeutic studies published in the literature are on HNSCC, and there is a dearth of studies on OSCC [130,131,132,133,134,135,136]. Hence, the oral cancer field avidly awaits the clinicians and researchers provision of more insights to maximize the potential of immunotherapies in future endeavors.

## Figures and Tables

**Table 1:** Cells of the TME and their biomarkers.

No.	Cell Type	Biomarkers	References
1	Macrophages	CD68 pan marker	Alves et al.; (2018) [47]
a.	M1 TAM	CD11c, CD80 and HLA–DR	Alves et al.; (2018) [47]
b.	M2 TAM	CD163, CD11b, CD206, MRC1	Alves et al.; (2018) [47]
2	DC	CD1a-immature, CD83-mature	Jardim et al.; (2018) [48]
3	NK	CD57	Taghavi et al.; (2016) [49]
4	MDSC	CD33 and CD11b	Pang et al.; (2020) [52]
5	T cells	CD3 pan marker	Olsen et al.; (2020) [1]
a.	Tregs	CD4^+^ CD25^+^ FOXP3^+^ and CD4^+^ CD25^+^ CD127^low^	Liu et al.; (2016) [34]
b.	Cytotoxic T cells	CD8 pan marker	Olsen et al.; (2020) [1]
6	B cells	CD19 and CD20 pan marker	Olsen et al.; (2020) [1]
7	CAF	α-SMA	Lao et al.; (2016) [51]
8	Endothelial cells	CD31, CD34	Teofilo et al.; (2020) [53]

## Data Availability

Not applicable.
